# Supplementary material for: Novel Selection Approaches to Identify Antibodies Targeting Neoepitopes on the C5b6 Intermediate Complex to Inhibit Membrane Attack Complex Formation
Source: Antibodies (Basel). 2021 Oct 12;10(4):39. doi: 10.3390/antib10040039 (PMC8544208; doi:10.3390/antib10040039)
Supplement: Supplementary file 1 [file antibodies-10-00039-s001.zip › Figures S1 and S2.pdf]

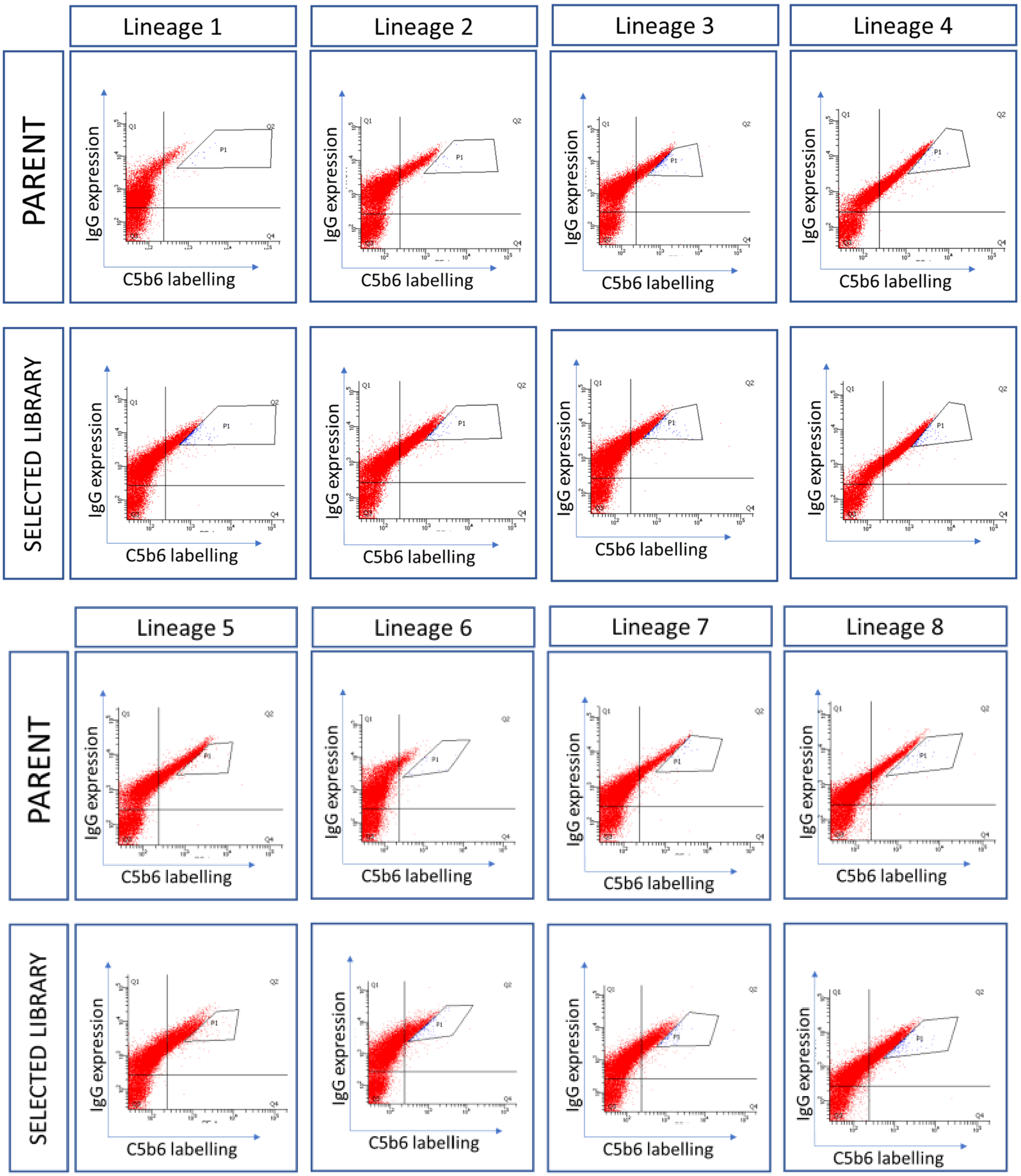

Supplementary Figure S1: Final round of CDRH1/CDRH2 diversified affinity maturation selections. The plots show the selected libraries compared with their corresponding parents. The gates drawn show ~0.1% of the selected library which were sorted prior to daughter clone expression. Six of the eight libraries show improved binding over parent, with lineages 3 and 5 showing no discernible improvement.

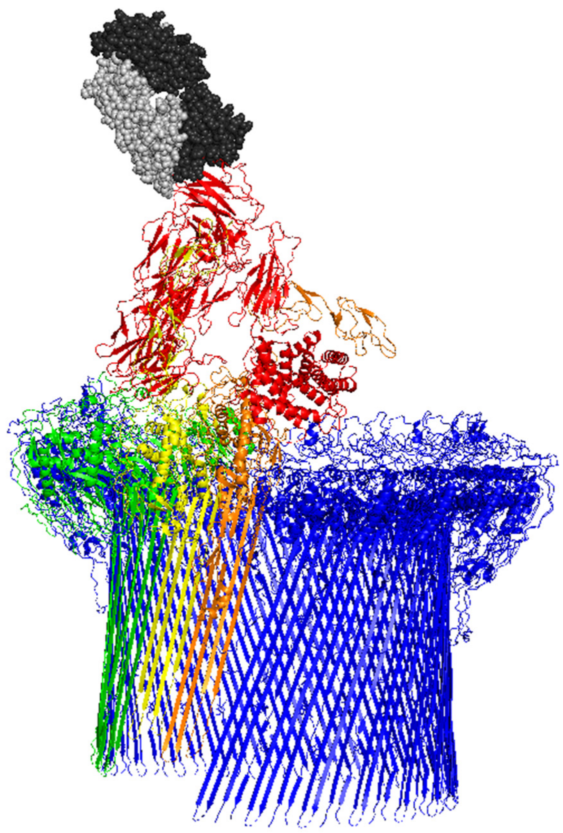

Supplementary Figure S2: Model of membrane attack complex (PDB: 6H04) and anti-C5 antibody (PDB: 5I5K) in complex with C5. Composite model generated and visualised using PyMol using structure of MAC (PDB: 6H04) and anti-C5 Fab in complex with C5 (PDB: 5I5K), by aligning both structures on the C5 chain. C5 (red), C6, (orange), C7 (yellow), C8 (green), C9 (blue), anti-C5 heavy chain (dark grey), anti-C5 light chain (light grey).
