# Supplementary material for: Novel Selection Approaches to Identify Antibodies Targeting Neoepitopes on the C5b6 Intermediate Complex to Inhibit Membrane Attack Complex Formation
Source: Antibodies (Basel). 2021 Oct 12;10(4):39. doi: 10.3390/antib10040039 (PMC8544208; doi:10.3390/antib10040039)
Supplement: Supplementary file 1 [file antibodies-10-00039-s001.zip › File S1.pdf]

Methods for modelling C5b6

All modelling and simulation was performed in MatLab SimBiology v5.2 The following simple model was used to describe the C5b6 complex formation and proposed antibody binding:

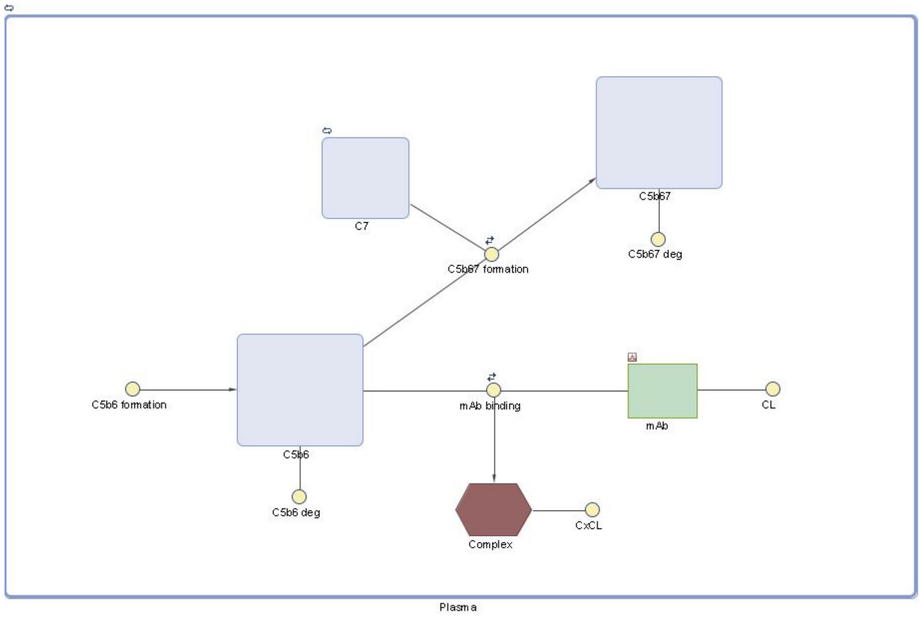

Figure 1: schematic outling simple model for C5b6-C7 binding in MatLab SimBiology.

The following assumptions were made:

- C7 concentration is 460nM and assumed to be at steady state due to high molar excess compared to C5b6
- The rate at which C5b6 forms is constant and known and there is no requirement to model the upstream interactions
- C5b67 concentration can be used as surrogate for the final MAC concentration (requires adjustment of molecular weight)
- Current model assume reactions occur in plasma only

A complete and comprehensive review of the complement cascade kinetics was compiled in 2009 by Korotaevskiy et al. In this paper, the authors reviewed and summarised the current literature and then proposed a model framework for the kinetics of the complement system. The table below shows a summary of kinetic parameters.

The overall affinity of C7 for the C5b6 complex has previously been reported to be 2pM with on and off rates of 4E5 M-1 s-1 and 8E-7 s-1 respectively6, and has been confirmed in house using Biacore (N30596-66, 68, 74, 80 and N3110-32). Note for all subsequent simulations the C7 affinity reported by Korotaevskiy (2009) was used (2pM).

The molecular weight of C5b67 is 179 (C5b) + 120 (C6) + 110 (C7) = 409 kDa, and the molecular weight of MAC is 305 (C5b67) + 152 (C8) + 8 * 93 (8 * C9 molecules on average) = 1201, therefore if we apply the conversion factor of 409 / 1201 = 0.34, we can adjust the molarity of C5b67 to that of MAC.

The model described in figure 1 was implemented in MatLab Simbiology v5.2 using parameters from the literature. Below are the ordinary differential equations (ODEs), fluxes, parameter values and starting conditions used in the simulation.

ODEs:

d(C5b6)/dt = 1/Plasma*(-ReactionFlux1 - ReactionFlux2 - ReactionFlux4 + ReactionFlux5)

d(C5b67)/dt = 1/Plasma*(ReactionFlux2 - ReactionFlux3)

d(mAb)/dt = 1/Plasma*(-ReactionFlux4 - ReactionFlux6)

d(Complex)/dt = 1/Plasma*(ReactionFlux4 - ReactionFlux7)

Fluxes:

ReactionFlux1 = (C5b6deg*C5b6)*Plasma

ReactionFlux2 = (KonC7*C5b6*C7)*Plasma-(KoffC7*C5b67)*Plasma

ReactionFlux3 = (C5b67deg*C5b67)*Plasma

ReactionFlux4 = (Kon*C5b6*mAb)*Plasma-(Koff*Complex)*Plasma

ReactionFlux5 = C5b6syn

ReactionFlux6 = (Kcl*mAb)*Plasma

ReactionFlux7 = (Kcx*Complex)*Plasma

Parameter Values:

C5b6deg = 6.9e-06 (1/hr)

KonC7 = 0.0004 (1/nM*s)

KoffC7 = 8e-07 (1/hr)

C5b67deg = 6.9e-06 (1/hr)

Kon = 0.01 (1/nM*s)

Koff = 1e-05 (1/hr)

C5b6syn = 1.5e-06 (nmole/s)

Kcl = 3.8e-07 (1/s)

Kcx = 6.9e-06 (1/s)

Plasma = 3 (L)

Initial Conditions:

C5b6 = 0

C5b67 = 0

mAb = 0

Complex = 0

C7 = 462 (nM)

A number of simulations were run exploring how the dose and the affinity impact the level of target engagement of C5b6. The aim was to achieve >90% target engagement of C5b6. The following simulations were run:

Simulation	Dose	Affinity (on, off rate)	Half Life	Max target engagement	Trough target engagement
1	10mg/kg (IV)	100pM (1E5 M^{-1} s^{-1} , 1E-5 s^{-1})	Typical	45%	8%
2	10mg/kg (IV)	10pM (1E6 M^{-1} s^{-1} , 1E-5 s^{-1})	Typical	89%	58%
3	10mg/kg (IV)	1pM (1E6 M^{-1} s^{-1} , 1E-6 s^{-1})	Typical	89%	75%
**4**	**10mg/kg (IV)**	**1pM (1E7 M^{-1} s^{-1} , 1E-5 s^{-1})**	**Typical**	**98%**	**93%**
**5***	**10mg/kg (IV)**	**1pM (1E7 M^{-1} s^{-1} , 1E-5 s^{-1})**	**Typical**	**93%**	**81%**

*Association rate constant for C5b6 reaction with C7 increased 5-fold, from 4E5 1/(M s) to 2E6 1/(M s) as reported by Thai and Ogata et al, 2005). Dissociation rate constant unchanged.

Simulations from rows 4 and 5 were plotted in figure 1 of the main text

A second round of simulations were performed with the constraints of a maximum 200mg subcutaneous (SC) dose. The simulations were repeated using this new fixed dose limit.

Simulation	Dose	Affinity (on, off rate)	Half Life	Max target engagement	Trough target engagement
5	200mg SC	1pM (1E7 M^{-1} s^{-1} , 1E-5 s^{-1})	Typical	94%	81%
6	200mg SC	0.1pM (1E7 M^{-1} s^{-1} , 1E-6 s^{-1})	Typical	97%	90%
7	200mg SC	1pM (1E7 M^{-1} s^{-1} , 1E-5 s^{-1})	YTE x2	95%	92%
8	200mg SC	1pM (1E7 M^{-1} s^{-1} , 1E-5 s^{-1})	YTE x3	96%	95%

Note that reducing the amount of drug from 10mg/kg IV (~700mg) to 200mg SC (~120mg IV assuming 60% bioavailability) has a minimal impact on the level of target engagement. This indicates that at 200mg Sc we are close to saturating the target.
